# Effect of zinc oxide nanoparticles on viability of human spermatozoa

**Published:** 2013-09

**Authors:** Abolfazl Barkhordari, Seyedhossein Hekmatimoghaddam, Ali Jebali, Mohammad Ali Khalili, Alireza Talebi, Marzieh Noorani

**Affiliations:** 1 *Department of Occupational Health, School of Health, Shahid Sadoughi University of Medical Sciences, Yazd, Iran.*; 2 *Department of Laboratory Sciences, School of Paramedicine, Shahid Sadoughi University ofMedical Sciences, Yazd, Iran.*; 3 *Department of Medical Nanotechnology, Pajoohesh Lab, Yazd, Iran.*; 4 *Research and Clinical Center for Infertility, Shahid Sadoughi University ofMedical Sciences, Yazd, Iran.*; 5 *Department of Occupational Health, School of Paramedicine, Abarkooh, Yazd, Iran*

**Keywords:** Spermatozoa, Viability, MTT assay, ZnO nanoparticles, Semen, Cytotoxicity.

## Abstract

**Background:** The extensive use of different nanoparticles has raised great concerns about their occupational and biological safety.

**Objective: **The aim of this study was to evaluate the cytotoxic effect of zinc oxide nanoparticles (ZnO NPs) on viability of spermatozoa.

**Materials and Methods:** Semen samples were obtained from 15 healthy persons, and were analyzed using WHO guidelines. Each semen sample was separately incubated with different concentrations of ZnO NPs (10, 100, 500, and 1000 µg/mL) at 37^o^C for 45, 90, and 180 minutes. Then, the cell death percentage of spermatozoa was measured by MTT assay. Mann-Whitney test was used for comparison of different times and concentrations.

**Results: **The maximum cell death percentage was 20.8%, 21.2%, and 33.2% after 45, 90, and 180 minutes, respectively. In case of concentration, the highest concentration (1000 µg/mL) of ZnO NPs led to the highest toxicity for all incubation times. Statistically, there were significant differences in cell viability after 180 minutes vs. 45 and 90 minutes.

**Conclusion:** This study indicated that cytotoxicity of ZnO NPs is dose and time dependent.

This article extracted from M.Sc. thesis. (Marzieh Noorani)

## Introduction

There are some occupational environments which may potentially affect fertility. Previous studies showed that some toxic chemicals, heavy metals, pesticides, and radiation can lead to infertility (1). Nanoparticles (NPs), with at least one dimension at 1-100 nm range, have become much prevalent in life over the past two decades, and are now widely used in a variety of industries and medical fields. These applications have raised concerns about their biological effects. 

It has previously been found that biological effects of nano-sized particles are quite different from their micro-scale particles. Interestingly, some studies have demonstrated that systemic sclerosis, rheumatoid arthritis, systemic lupus erythematous, and chronic renal disease are associated with exposure to NPs (2). Although the effects of environmental and occupational particles on human fertility are well documented, but there are few studies about the effects of NPs on sperms. For example, it has been recorded that iron oxide NPs (Fe_3_O_4_) have no effect on sperm motility (3). 

According to Wiwanitkit *et al* study, sperm motility was decreased (25%) after exposure to gold NPs, compared with normal group (95%) (4). Ema *et al* revealed that titanium dioxide (TiO_2_) NPs decrease human sperm motility, and also silica NPs are toxic for mouse sperms (5). In 2011, a study on toxicity of TiO_2_ and silver NPs on reproductive system was done by Philbrook *et al*. Their results showed that a single oral 100 mg/kg dose of TiO_2_ NPs decreased mice developmental process, and increased fetal deformities and mortality (6). There are no studies on impact of Zinc oxide (ZnO) NPs on sperm. Some researchers have declared toxicity of ZnO NPs on different cells. 

For example, we know that ZnO NPs can damage alveolar epithelial cells in a dose- and time-dependent manner, and may cause mitochondrial dysfunction, as they can increase intracellular reactive oxygen species (ROS) (7). Toxicity of ZnO NPs on human bronchial epithelial cells was investigated, and suggested that oxidative stress is a mechanism of toxicity (8). Also, it was demonstrated that ZnO NPs inhibit growth of Staphylococcus aureus, Pseudomonas aeruginosa, and Saccharomyces cerevisiae (9){Heng, 2010 #19}. The aim of this study was to investigate the cytotoxic effects of ZnO NPS on human spermatozoa using MTT assay.

## Materials and methods

**Materials**

ZnO NPs were purchased from Lolitech Co., Germany. RPMI (Roswell Park Memorial Institute) 1640, 70% isopropanol, and 3-(4, 5-dimethylthiazol-2-yl)-2, 5-diphenyl-tetrazolium bromide (MTT) were obtained from Sigma-Aldrich Chemical Co., (St Louis, MO). Hanks’ balanced salt solution (HBSS) was provided from Gibco Invitrogen, UK.

**Preparation and characterization of ZnO NPS**

In this laboratory trial, different concentrations of ZnO NPs (10, 100, 500, and 1000 µg/mL) were prepared in RPMI 1640, and gently shaken for 5 min. The structure of ZnO NPs was characterized by scanning electron microscopy (SEM) (Hitachi S-2400) at 15 KV as accelerating voltage, and their size distributions were analyzed by dynamic light scattering (DLS) (Malvern Instruments, Italy).

**Preparation of sperms and nanoparticle exposure**

For statistical testing purposes, 15 normal fresh semen samples from healthy persons were obtained and analyzed using WHO guidelines (10). They were 20-40 years old men examined by specialists in urology for being healthy regarding any disease which may affect spermatogenesis, and signed an informed consent form. All were husbands of women referred for treatment of female infertility. This study was in compliance with the ethics committee of Shahid Sadoughi University of Medical Sciences, Yazd, Iran. In the first step, 10 µL of ejaculated semen was obtained after 2-4 days of abstinence from intercourse, and washed three times with buffered normal saline. Washed sperms were resuspended in RPMI 1640 and adjusted at 5×10^3^ cells/mL. 

Then, 100 µL of each sample was added to separate wells in a 96-well microplate. In the next step, 100 µL of different concentrations of ZnO NPS was added to sperm samples, and incubated at 37^o^C for 45, 90, and 180 minutes. As negative control, sperms were not treated with ZnO NPs. But, as positive control, sperms were exposed to 0.1 M HCl. Same as the treated samples, both negative and positive control samples were incubated at 37^o^C for 45, 90, and 180 minutes, too.

**MTT assay**

The cytotoxicity of NPs was evaluated by MTT assay. The reduction of MTT salt to Formosan crystal by mitochondrial dehydrogenase enzymes is the mechanism of this assay (11). After incubation, sperm cells were washed three times by HBSS, and 10 µL of 5 mg/mL MTT solution was added to each well. After 3 hours incubation at 37^o^C, 50 µL of 70% isopropanol was added, and optical density (OD) of each well was read at 490 nm using a micro plate reader (Novin Gostar, Iran). Finally, the cell death percentage of each group was measured, according to below formula (12).

$\mathrm{Sperm death}\left(\%\right)=A-B/A\times 100$

Where

A is the OD of negative control,

B is the OD of sample after exposure to ZnO NPs

**Statistical analysis**

Toxicity of each concentration of ZnO NPs was evaluated twice, and its average was calculated. Statistical analysis was performed by SPSS v.16 software (SPSS Inc, Chicago, USA). Mann-Whitney test was used for detection of significant differences. Any P-value less than 0.05 were considered as statistically significant difference.

## Results

**Figure 1:**
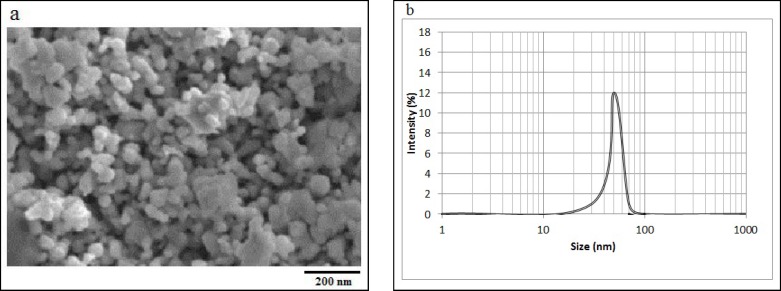
The SEM image of ZnO NPs (a), and the size distribution of ZnO NPs (b), obtained by DLS method

**Figure 2:**
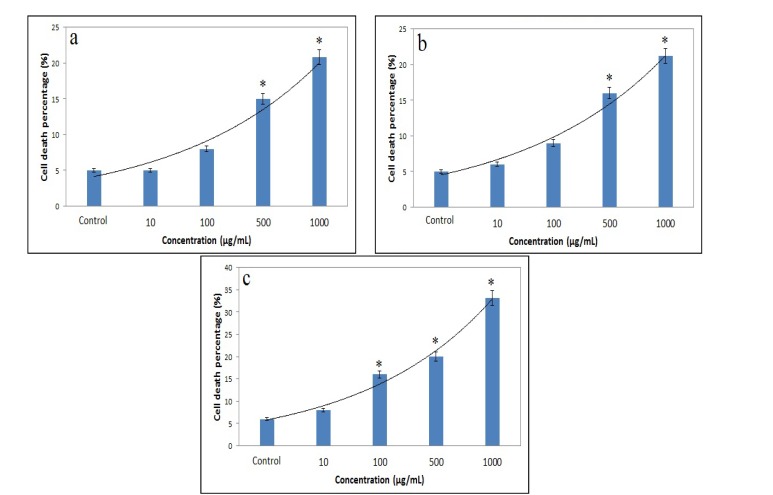
The cell death percentage after treatment with ZnO NPs.

The SEM image of ZnO NPs is shown in figure 1a. This image shows that the structure of ZnO NPs is amorphous, and their sizes are about 50 nm. As shown in figure 1b, the size distribution of ZnO NPs is about 30-70 nm. The relationships between different concentrations of ZnO NPs and cell death percentage after 45, 90, and 180 minutes are presented in figure 2a, figure 2b, and figure 2c, respectively. 

According to these figures, the maximum cell death after 45, 90, and 180 minutes was 20.86%, 21.2%, and 33.26%, respectively. In all incubation times, the highest cell death was observed at concentration of 1000 µg/mL, and the lowest one at 10 µg/mL. Based on figures 2a, 2b and 2c, with increase in incubation time from 45-90 min, no significant change was observed in cell death, but after 180 minutes exposure, the percentage of cell death was increased at all concentrations. This research showed that at all incubation times, there are significant differences between cell death related to ZnO NPs at concentration of 500 µg/mL and 1000 µg/mL vs. control group (p=0.001). On the other hand, significant differences were shown between cell death percentage after 180 minutes vs. 45 and 90 minutes (p=0.001) at concentration of 100, 500, and 1000 µg/mL. However, there was no such difference between cell death at 45 min and 90 minutes (p=0.08).

At first, 100 µL of spermatozoa suspension was separately incubated with different concentrations of ZnO NPs at 37^º^C for 45 (a), 90 (b), and 180 (c) minutes. Then, spermatozoa were washed by HBSS, and 10 µL of 5 mg/mL MTT solution was added. After 3 hours incubation, OD of each well was read at 490 nm. Finally, the cell death percentage of each sample was calculated, according to formula 1. All data are shown as mean±SD. *p<0.05 compared with control.

## Discussion

To evaluate spermatotoxicity of ZnO NPs, different concentrations of these NPs (10,100, 500 and 1000 µg/mL) were prepared and added to sperm cells for 45, 90, and 180 minutes. In this study, the percentage of cell death was measured by MTT assay. According to our results, when cells were treated with higher concentrations of ZnO NPs, their toxicity was increased. This means that ZnO NPs affect sperm cells in a dose-dependent manner. 

Also, this research showed that the maximum cell death was 20.86%, 21.2%, and 33.26% after 45, 90, and 180 minutes, respectively. As shown in figure 2a, 2b and 2c, the toxicity to sperms is increased with increase in incubation times. This clearly suggests a time-dependent process of toxicity. Dose-dependent and time-dependent toxicity of NPs are commonly seen in nanotoxicological studies (-). As an important finding extracted from the present study, although there were little increments in cell death after 90 minutes, but a high rise (33.26%) was observed after 180 minutes. Compared with silver and TiO_2_ NPs which have high toxicity, ZnO NPs have moderate toxicity on sperms. 

Although there is no study on toxic effect of ZnO NPs on human sperm cells, but toxicity of other NPs were investigated in previous studies. For example, Ema *et al* showed that silica NPs are toxic for spermatozoa at high concentrations (5). In a recent study, it was shown that gold NPs decrease sperm motility, and TiO_2_ NPs are more cytotoxic for Leyding cells than other NPs (4, 12). In addition, previous studies have mainly focused on changes in the motility of sperm cells (13-15). According to previous researches, ZnO NPs damage alveolar epithelial cells in a dose- and time-dependent manner (7). Also, ZnO NPs can affect human bronchial epithelial cells (8). The toxicity mechanisms of ZnO NPs have not been well defined. Mitochondrial dysfunction, ion release, binding to membrane or cytosol protein, and generation of intracellular ROS are some of proposed mechanisms of toxicity (8).

In this study, MTT assay was used to evaluate cytotoxicity. The MTT assay is a simple, rapid and reliable method for estimation of percentage of cell viability, depending on accumulation of Formosan crystals (16, 17). As noted before, mitochondrial dehydrogenase enzymes reduce MTT salt to Formosan. We used only MTT test, and this is the main limitation of this study. Other tests such as release of lactate dehydrogenase, generation of ROS, annexin dye binding, and investigation of involved genes should be used in future studies. Toxicity of ZnO NPs would also be investigated in actual conditions. The authors suggest an in vivo study (both in animal models and exposed patients) for toxicity evaluation of ZnO NPs. Taken together; ZnO NPs have spermatotoxicity, and may lead to infertility after exposure. Toxicity of ZnO NPs is dependent on both concentration and exposure time.
